# Metal availability shapes early life microbial ecology and community succession

**DOI:** 10.1128/mbio.01534-24

**Published:** 2024-10-23

**Authors:** Joshua Soto Ocaña, Elliot S. Friedman, Orlaith Keenan, Nile U. Bayard, Eileen Ford, Ceylan Tanes, Matthew J. Munneke, William N. Beavers, Eric P. Skaar, Kyle Bittinger, Babette S. Zemel, Gary D. Wu, Joseph P. Zackular

**Affiliations:** 1 Division of Protective Immunity, Children’s Hospital of Philadelphia, Philadelphia, Pennsylvania, USA; 2 Department of Pathology and Laboratory Medicine, Perelman School of Medicine, University of Pennsylvania, Philadelphia, Pennsylvania, USA; 3 Division of Gastroenterology & Hepatology, Perelman School of Medicine, University of Pennsylvania, Philadelphia, Pennsylvania, USA; 4 Division of Gastroenterology, Hepatology and Nutrition, The Children’s Hospital of Philadelphia, Philadelphia, Pennsylvania, USA; 5 Department of Pediatrics, University of Pennsylvania Perelman School of Medicine, Philadelphia, Pennsylvania, USA; 6 Department of Pathology, Microbiology and Immunology, Vanderbilt University School of Medicine, Nashville, Tennessee, USA; 7 The Center for Microbial Medicine, Children’s Hospital of Philadelphia, Philadelphia, Pennsylvania, USA; University of Maryland School of Medicine, Baltimore, Maryland, USA

**Keywords:** gut microbiome, heavy metals, microbial ecology, human microbiome, early life, neonates

## Abstract

The gut microbiota plays a critical role in human health and disease. Microbial community assembly and succession early in life are influenced by numerous factors. In turn, assembly of this microbial community is known to influence the host, including immune system development, and has been linked to outcomes later in life. To date, the role of host-mediated nutritional immunity and metal availability in shaping microbial community assembly and succession early in life has not been explored in depth. Using a human infant cohort, we show that the metal-chelating protein calprotectin is highly abundant in infants. Taxa previously shown to be successful early colonizers of the infant gut, such as *Enterococcus*, *Enterobacteriaceae,* and *Bacteroides,* are highly resistant to experimental metal starvation in culture. *Lactobacillus*, meanwhile, is highly susceptible to metal restriction, pointing to a possible mechanism by which host-mediated metal limitation shapes the fitness of early colonizing taxa in the infant gut. We further demonstrate that formula-fed infants harbor markedly higher levels of metals in their gastrointestinal tract compared to breastfed infants. Formula-fed infants with high levels of metals harbor distinct microbial communities compared to breastfed infants, with higher levels of *Enterococcus*, *Enterobacter,* and *Klebsiella*, taxa which show increased resistance to the toxic effects of high metal concentrations. These data highlight a new paradigm in microbial community assembly and suggest an unappreciated role for nutritional immunity and dietary metals in shaping the earliest colonization events of the microbiota.

**IMPORTANCE:**

Early life represents a critical window for microbial colonization of the human gastrointestinal tract. Surprisingly, we still know little about the rules that govern the successful colonization of infants and the factors that shape the success of early life microbial colonizers. In this study, we report that metal availability is an important factor in the assembly and succession of the early life microbiota. We show that the host-derived metal-chelating protein, calprotectin, is highly abundant in infants and successful early life colonizers can overcome metal restriction. We further demonstrate that feeding modality (breastmilk vs formula) markedly impacts metal levels in the gut, potentially influencing microbial community succession. Our work suggests that metals, a previously unexplored aspect of early life ecology, may play a critical role in shaping the early events of microbiota assembly in infants.

## INTRODUCTION

The human gastrointestinal (GI) tract is inhabited by a diverse collection of microorganisms that aids in digestion, stimulates the immune system, and provides essential vitamins and nutrients to the host (1). Perturbations to the early life microbiota alter the maturation of this microbial community and impact its relationship with the host (2). Disruption of the gut microbiota early in life negatively impacts host health by modulating immune cell development and tolerance, leaving the host prone to inflammatory diseases, such as allergic and atopic diseases, later in life (2–6). However, the processes governing gut microbiota succession during early life, including host and environmental factors, remain unclear.

Newborns are rapidly colonized by microbes following birth. Assembly of this community at this early time point is impacted by delivery mode. Infants that are born via vaginal birth harbor distinct microbiomes from those born via cesarian section (c-section) (7, 8). Discrepancies between the birthing method and early life microbiota composition are thought to be driven by the fact that babies born via vaginal birth are exposed to the maternal vaginal microbiota. Vaginally delivered infants commonly harbor communities similar to the mother’s vaginal microbiome, whereas c-section infants harbor communities similar to the skin microbiota (7, 8). Vaginally born infants have higher levels of bacteria from the genera *Bacteroides*, *Bifidobacterium*, and *Escherichia* (7, 8). Surprisingly, in many infant cohorts, vaginal delivery is not correlated with colonization by or levels of members of the genus *Lactobacillus* (9, 10), one of the most abundant genera of the maternal vaginal microbiota (11). The underlying reason for this lack of *Lactobacillus* representation in babies is not well understood, but likely involves undefined ecological factors shaping fitness and colonization in the neonatal gut ecosystem. In the ensuing months following birth, differences in the gut microbiota between c-section and vaginally born infants decrease as the microbial community develops and overall diversity increases.

Community succession and structure during early life are impacted by feeding practices, as breast and formula feeding drive distinct community structures (12, 13). Breastfed infants are dominated by members of the genus *Bifidobacterium (13*), whereas formula-fed infants harbor microbial communities that resemble older children (12, 13). These differences in breastfed infants have been shown to be driven by different factors, including the presence of breastmilk oligosaccharides that select for organisms that metabolize complex sugars, maternal IgA that prevents expansion of pathogenic bacteria, and antimicrobial peptides and proteins (14–16). As infants develop, added exposure to environmental microorganisms and a transition from breastmilk- or formula-based liquid diets to solid foods promote further maturation of the microbiota (3, 17).

Beyond milk-derived nutrients, little is known about the nutritional landscape shaping early life succession of the microbiota in the first year of life. One important micronutrient for all of life are the transition metals (18). Metals are trace nutrients that are essential for the survival of all living cells. Metals play roles in biochemical and enzymatic processes, and are used as co-factors in metabolism, respiration, detoxification, and DNA transcription reactions (19). Dietary metals are absorbed by the host in the GI tract and influence the ecology of and competition within the gut microbiota (20). Commensal microbes and bacterial pathogens utilize metals for metabolic processes needed for survival. This need for metals is exploited by the host to limit pathogen fitness during infection through the production of metal-sequestering proteins, like calprotectin, in a process termed nutritional immunity (18, 21, 22). Surprisingly, little is known about the role of nutritional immunity and interspecies competition for metals on the gut microbiota and nutritional landscape in the gut. Dietary metal levels can impact bacterial infections and can shape the bacterial gut microbiota, leaving the host prone to colonization with bacterial pathogens (23). Despite growing appreciation for the role of metals in the gut, little is known about the impact of metal availability in shaping the early life microbiota.

In a previous study using a human infant cohort, we showed infants harbor high levels of human-derived proteins, specifically high levels of the calprotectin family of proteins which accounted for ~6% of the total protein in stool contents (24). We also showed that at 1 month of life infant gut microbial communities have high levels of members of the genus *Bifidobacterium*, *Enterococcus*, *Klebsiella*, *Enterobacter*, and *Staphylococcus* (24). Moreover, in a separate study, calprotectin levels were shown to be elevated early in life and had an impact on gut microbiota composition (25). However, the mechanisms underlying microbial assembly and succession in the gut prior to 1 month of age are poorly understood. In this study, we sought to understand drivers of microbial community assembly and succession during infancy. We show that levels of the metal-chelating protein calprotectin are elevated at birth and during the first month of life. We used *in vitro* culture assays to determine the effects of metal limitation on the growth of strains from three groups of early life colonizers (*Enterococcus*, *Enterobacteriaceae*, and *Bacteroides*), as well as the most abundant genera from the human vaginal microbiome, *Lactobacillus*. We show that Lactobacillus are sensitive to metal limitation, while other early life colonizers, including *Bacteroides*, *Enterococcus*, and *Enterobacteriaceae* persist in metal-limited environments. This suggests that host-mediated nutritional immunity may support early life colonizers through the modulation of metal levels. We further demonstrate that feeding modality has a robust impact on the nutritional landscape of the gut, with formula-fed infants harboring markedly higher levels of metals in their stool. Increased metal levels in formula-fed babies were associated with a greater abundance of bacterial genera that are resistant to the toxic effect of excess metals. This suggests a previously unappreciated role for feeding modality in shaping the nutritional landscape of the infant gut and may explain differences seen in the early life microbiota. Taken together, this work provides new insights into the nutritional factors impacting microbial succession during early life and highlights the underappreciated role of nutritional immunity and dietary metals during this important developmental window.

## RESULTS

### The metal-chelating protein, calprotectin, is elevated in stool in early life

**Fig 1:**
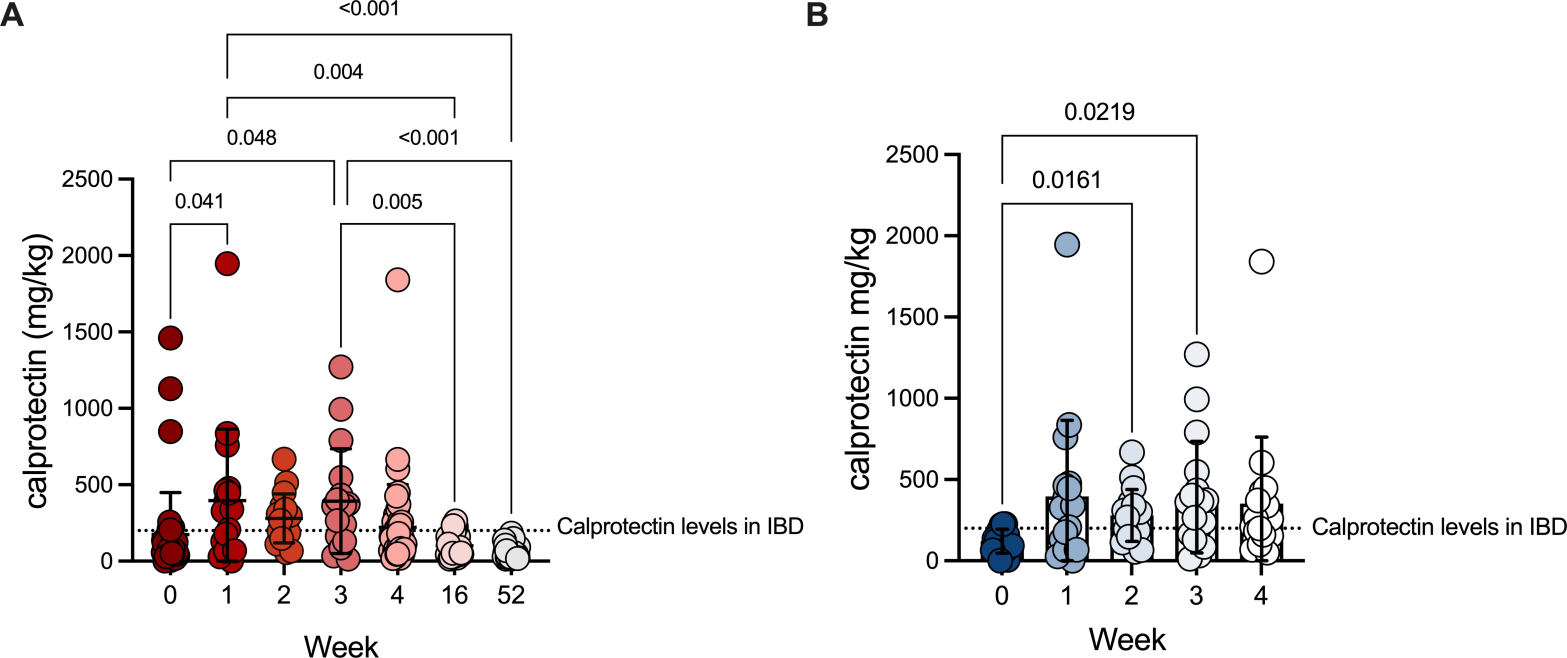
Fecal calprotectin, a metal-chelating protein, is elevated early in life. Calprotectin measured via ELISA in human stool samples from two sub-studies: 17 infants sampled weekly from birth (week 0) through 1 month of age (week 4) (sub-study 1), and 30 infants sampled at birth, 1 month, 4 months (week 16), and 1 year (week 52) of age (sub-study 2). Data are shown for (**A**) both sub-studies at all time points, and (**B**) sub-study 1 only from birth to 1 month. Significance was assessed by Tukey’s or Dunnett’s multiple comparisons tests. Adjusted *P* value < 0.05 considered statistically significant. (Error bars indicate mean with SD)

Environmental and host factors that shape microbiota colonization dynamics during early life are still incompletely understood. Metals are important nutrients that are essential for all forms of life and the balance of metals is critical at the host-microbe interface. Calprotectin is heterodimeric metal-chelating S100 protein that can sequester zinc, manganese, and iron (26). This important neutrophilic protein is associated with inflammation and inflammatory disorders in the intestines (27, 28) and increased fecal calprotectin is a biomarker for inflammatory bowel disease (IBD) (29). Calprotectin is also critical across a diversity of tissues for the control of numerous pathogens including *Staphylococcus aureus*, *Acinetobacter baumannii*, *Candida albicans*, *Clostridioides difficile*, and *Helicobacter pylori* (21). Interestingly, infants harbor high levels of calprotectin without manifesting any signs of inflammatory-associated diseases (30). In the context of nutritional immunity, metal chelation by calprotectin has antimicrobial activity against bacterial pathogens and commensals. However, the role of calprotectin on bacterial community assembly early in life has not been explored. In our previous work, we found that calprotectin family of proteins accounted ~6% of the total human protein content in infant stool (24). To examine this further, we quantified longitudinally the levels of calprotectin in infants from two sub-studies within the Infant Growth and Microbiome (IGraM) study (Fig. 1). IGraM is a prospective longitudinal cohort study of infant growth in the first 2 years of life in babies born to African American mothers conducted at the Children’s Hospital of Philadelphia. We quantified calprotectin levels in 17 infants weekly from birth (week 0) through 1 month of age (week 4) in sub-study 1, as well as in 30 infants at birth, 1 month, 4 months (week 16) , and 1 year (week 52) of age in sub-study 2. Taken together, the data from these independent, but integrated cohorts showed that, similar to previous reports (30–33), calprotectin levels increased following birth and remained elevated during the first month of life (Fig. 1A). Levels of calprotectin in these infants were well above the threshold used to diagnose inflammatory bowel disease in adults (34). Levels then decreased at 4 months and reached sub-threshold levels by 1 year (Fig. 1A). Within the first month of life, calprotectin levels increased following birth (Fig. 1B), concurrent with microbial colonization of the GI tract. Interestingly, calprotectin levels at birth are negatively correlated with gestational age (Fig. S1A), which suggests that elevated calprotectin levels early in life may be related to the maturation of the GI tract. As calprotectin levels are typically associated with intestinal inflammation in adults, we looked for correlations between fecal calprotectin levels and markers of intestinal inflammation at 1 month. Surprisingly, fecal calprotectin levels were not correlated with C-reactive protein, tumor necrosis factor (TNF) alpha, interleukin 6 (IL-6), or interleukin-1 beta (IL-1B) (Fig. S1B through E). These data suggest that elevated calprotectin levels early in life are not related to intestinal inflammation, as in adults, but rather may be associated with maturation and development of the GI tract.

**Fig 2:**
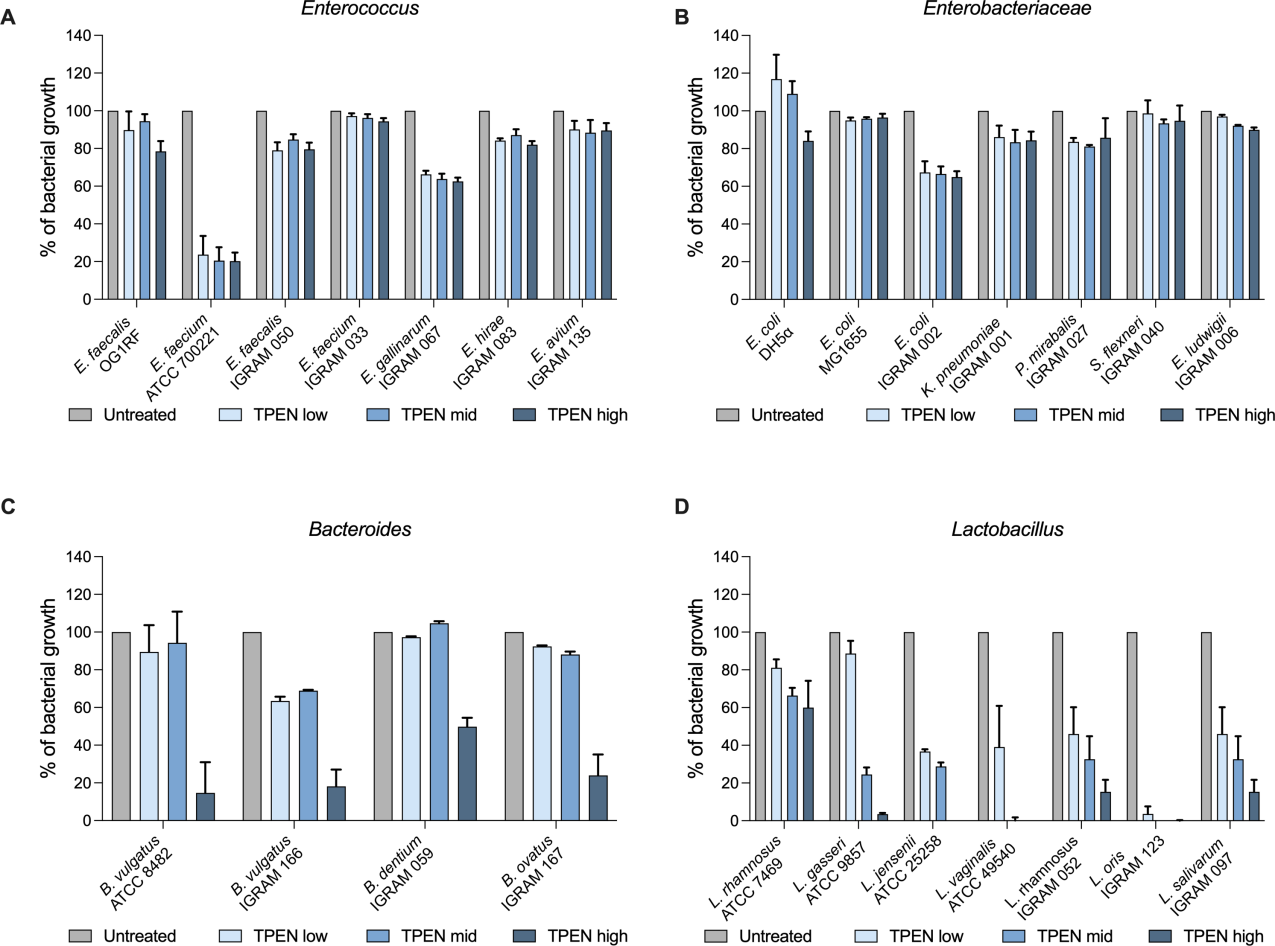
Metal levels impact growth of early life colonizing bacteria. (**A–D**) *In vitro* growth at 10 h of *Enterococcus*, *Enterobacteriaceae*, *Bacteroides*, and *Lactobacillus* strains in BHIS/MRSB treated with 50 µM TPEN (TPEN low), 60 µM TPEN (TPEN mid), or 70 µM TPEN (TPEN high) (*n* = 6) (Error bars indicate mean with SD).

To directly test the impact of metal availability on the fitness of early life colonizing taxa, we built a representative microbial cultivar library using publicly available reference strains as well as strains isolated from the IGraM infant cohort to test how representative early life colonizers responded to metal-limited conditions. To model the role of metal chelation by calprotectin, we used *N*,*N*,*N′*,*N′*-tetrakis (2-pyridinylmethyl)−1,2-ethanediamine (TPEN), a non-selective heavy metal chelator that has a similar chelating profile as calprotectin (35). We observed that *Enterococcus* and *Enterobacteriaceae* strains were highly resistant to metal chelation by TPEN (Fig. 2A and B). Members of the *Bacteroides* genus were moderately resistant to metal limitation, growing well in low-to-mid levels of TPEN (Fig. 2C). Notably, we observed that species from the *Lactobacillus* genus grew poorly in metal-depleted conditions and this group of microbes were highly sensitive to low levels of TPEN (Fig. 2D). These data suggest that metal availability, interspecies metal competition, and host-mediated metal restriction may differentially impact the fitness of early life colonizing microbiota.

### Taxa resistant to metal depletion *in vitro* are highly abundant during the first month of life when calprotectin levels are elevated

**Fig 3:**
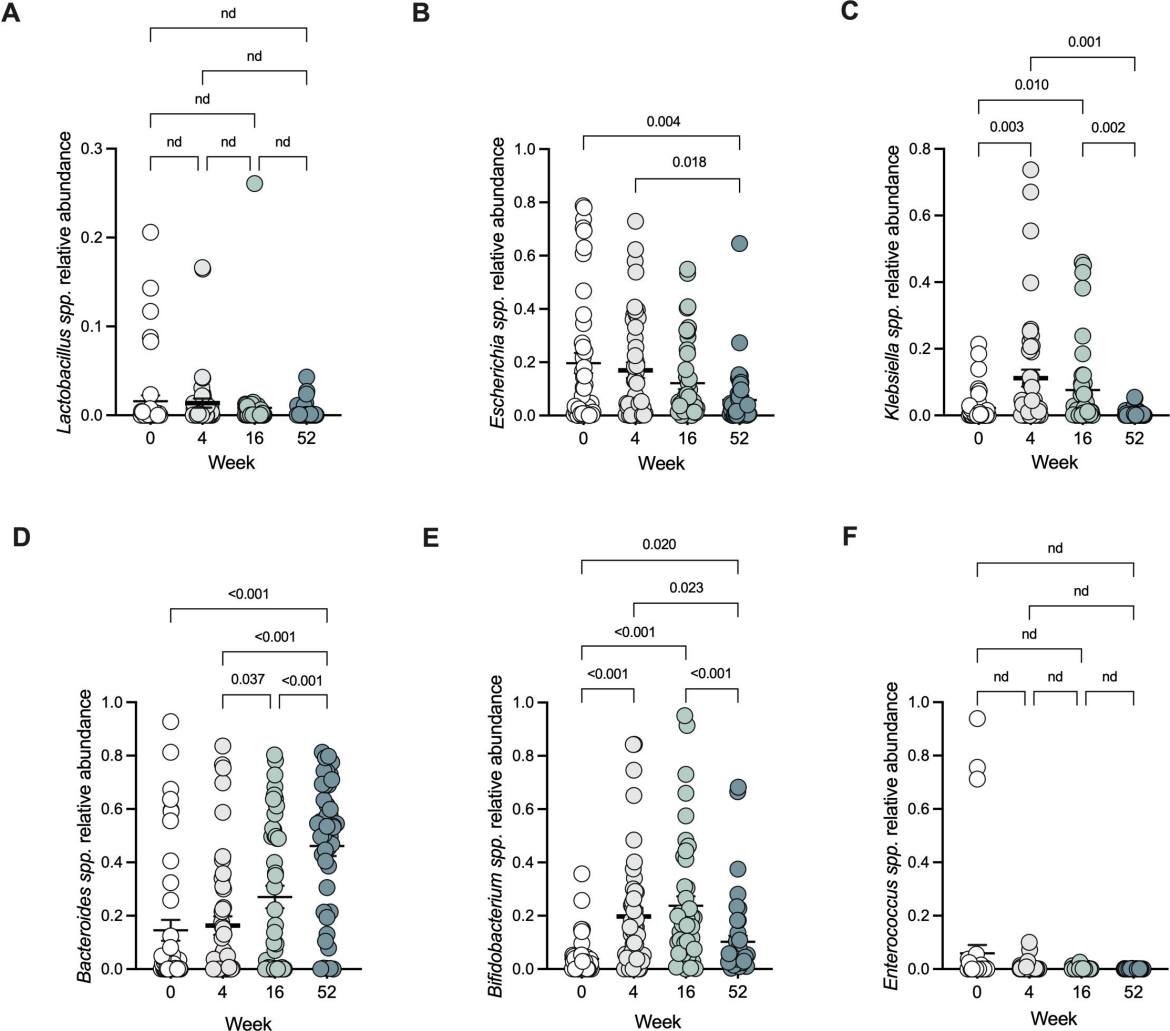
Taxa that are resistant to metal restriction are highly abundant during the first month of life when calprotectin levels are high. (**A–F**) Metagenomic analyses of *Lactobacillus*, *Escherichia*, *Klebsiella*, *Bacteroides*, *Bifidobacterium*, and *Enterococcus* relative abundances in infant human stool samples (*n* = 47). Paired *t*-tests with false discovery rate corrections for multiple comparisons were used with fdr <0.05 considered statistically significant. (Error bars indicate mean with SD)

During vaginal birth, infants are inoculated with maternal microbes that inhabit the maternal birthing canal. Interestingly, these infants are poorly colonized with the most abundant members of this microbial community, species from the genus *Lactobacillus*. In infants from our sub-study 1 cohort, we observed that reads affiliated with the *Lactobacillus* genus significantly decreased following birth (Fig. S2A), supporting the model that Lactobacilli are poor colonizers of the infant gut. This colonization defect is likely not associated with the inoculum, as birthing mothers harbored high levels of *Lactobacillus* in their vaginal microbiomes (Fig. S3). Other taxa of interest did not exhibit significant changes during the first month of life (Fig. S2B through F), likely due to limitations in the small sample size of this sub-study (*n* = 17). Thus, we combined reads from sub-study 1 and sub-study 2 to create an integrated cohort with taxonomic data at birth, 1 month, 4 months, and 1 year of age (*n* = 47). Analysis of the microbiota in this integrated cohort showed that taxa that were resistant to metal restrictions *in vitro* were elevated early in life (Fig. 3). Specifically, *Escherichia*, which we have previously shown to be a frequent early colonizer (24), remains elevated at 1 month and then decreases at 4 and 12 months (Fig. 3B), corresponding with the decrease in calprotectin levels. *Klebsiella,* which is present at low levels at birth, increases significantly at 1 and 4 months of age and then decreases at 12 months, also corresponding with the decrease in calprotectin levels (Fig. 3C). *Bacteroides* and *Bifidobacterium* increase in a time-dependent manner following birth, with *Bifidobacterium* decreasing at 12 months likely due to the transition to a complex solid diet (Fig. 3D and E). Interestingly, *Enterococcus* abundances did not change over the first year of life (Fig. 3F). These data demonstrate a stark variability in the fitness of various taxa in the early life infant gut ecosystem.

**Fig 4:**
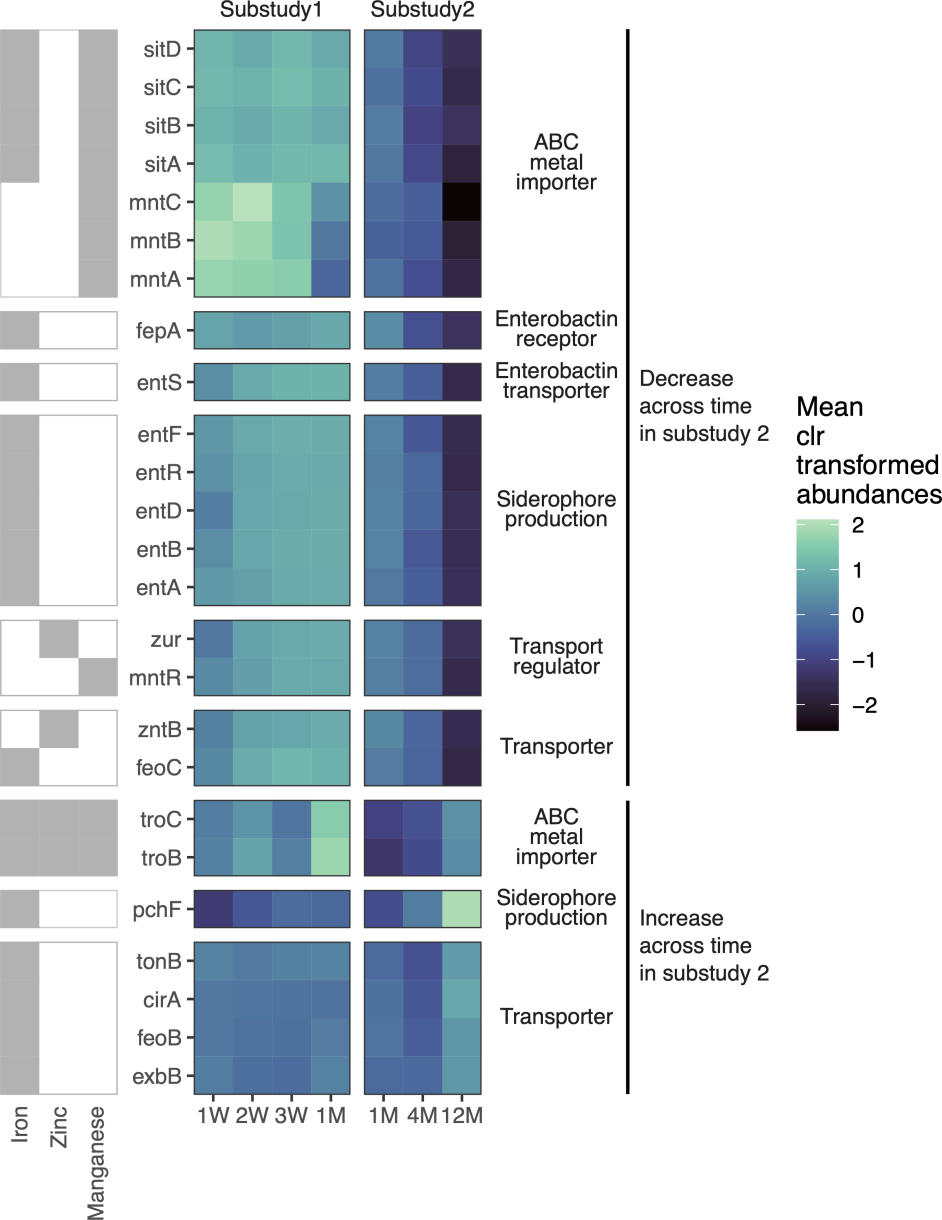
Metal import genes are time-dependent during early life. Heatmap showing mean clr transformed abundance of metal import genes in infants that had significant time effects in sub-study 2 (linear mixed-effects modeling, fdr <0.05). Within sub-study 1, only *mntA*, *mntB*, and *mntC* had significant time effects (linear mixed-effects modeling, fdr <0.05). Key represents which metals the genes interact with according to KEGG (gray = yes, white = no).

Based on these observations, we next sought to define gene networks that are associated with metal metabolism in the infant microbial community. We observed that the majority of metal import genes were elevated during the first month of life and then decreased significantly between 1 month and 1 year (Fig. 4). This result is consistent with the notion that the selective pressure of calprotectin and metal limitation leads to an increased representation of metal import genes in the gut microbiome of infants as an adaptive modality to increase fitness. These data suggest that nutritional immunity may reshape the landscape of the early life ecosystem and likely influences microbial community composition and assembly during this critical window of development.

### Dietary metals impact metal levels in the gut and bacterial community composition during early life

**Fig 5:**
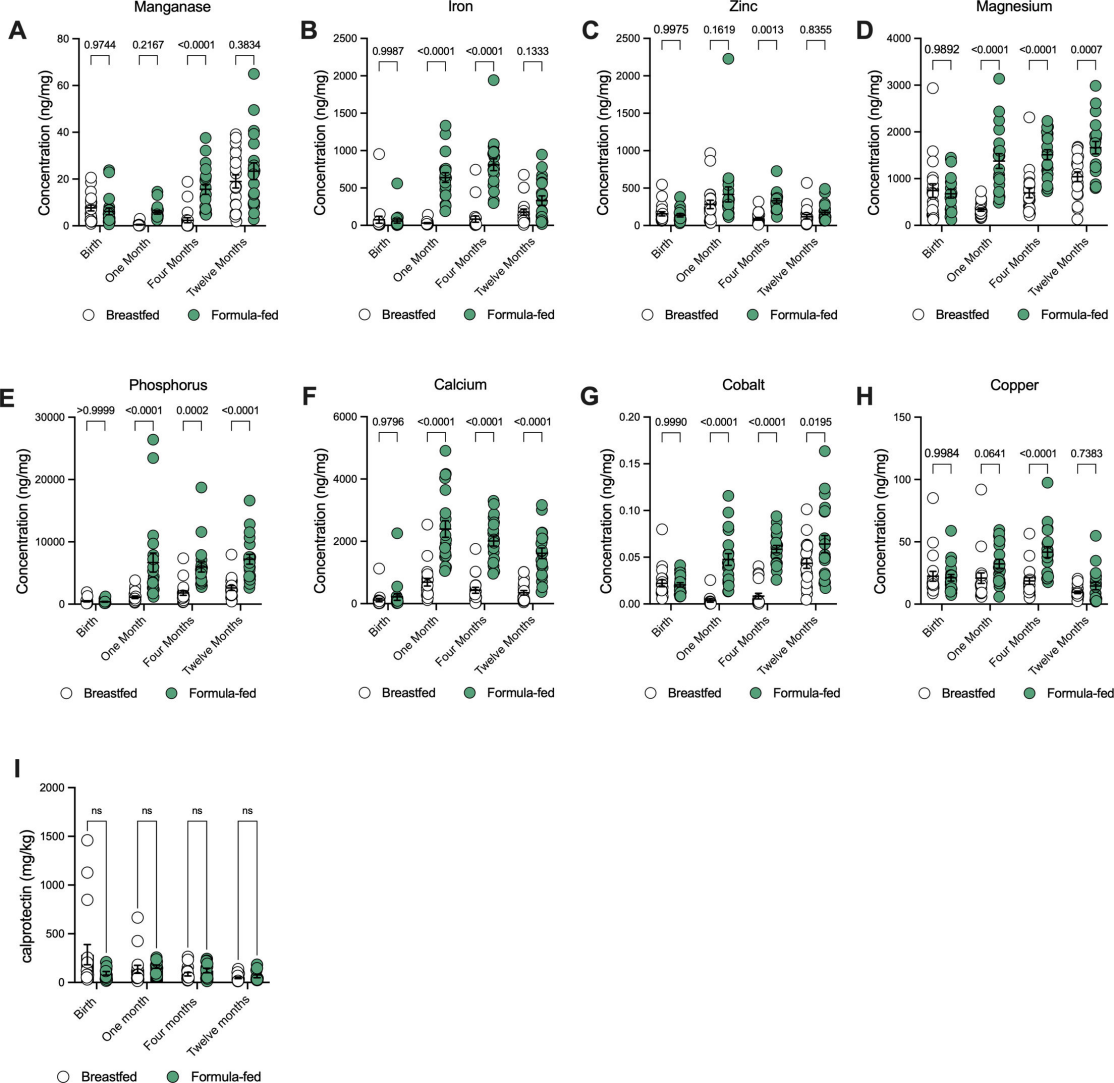
Formula-fed babies harbor increased levels of metals in the gut during early life. (**A–I**) Elemental metals quantification via ICP-MS in human infant stool samples (*n* = 20 per group, two-way ANOVAs with Geisser-Greenhouse corrections). (**J**) Calprotectin measurements in human stools measured via ELISA (*n* = 17 for breastfed and *n* = 13 for formula-fed). ANOVA with Geisser-Greenhouse correction was performed to assess statistical significance.

To further assess the role of metals and metal chelation during early life, we quantified metal levels in the stool of babies during the first year of life. Surprisingly, we observed a strong partitioning of metal levels in stool based on feeding type. Formula-fed infants harbored significantly higher levels of metals in stool compared to breastfed infants. We observed this marked increase specifically in manganese, iron, zinc, magnesium, phosphorus, calcium, cobalt, and copper (Fig. 5A through H). Potassium, sodium, sulfur, selenium, and chromium did not differ between breast- and formula-fed infants (Fig. S3). These data suggest that the metal content of infant formula dramatically alters the nutritional landscape in the gut of infants. To determine if the differences in levels of metals were impacted by the chealating effects of calprotectin, we analyzed calprotectin levels in formula- and breastfed infants from sub-study 2 across the first year of life. Here, we observed no significant differences in calprotectin levels at 1, 4, and 12 months of life (Fig. 5I).

**Fig 6:**
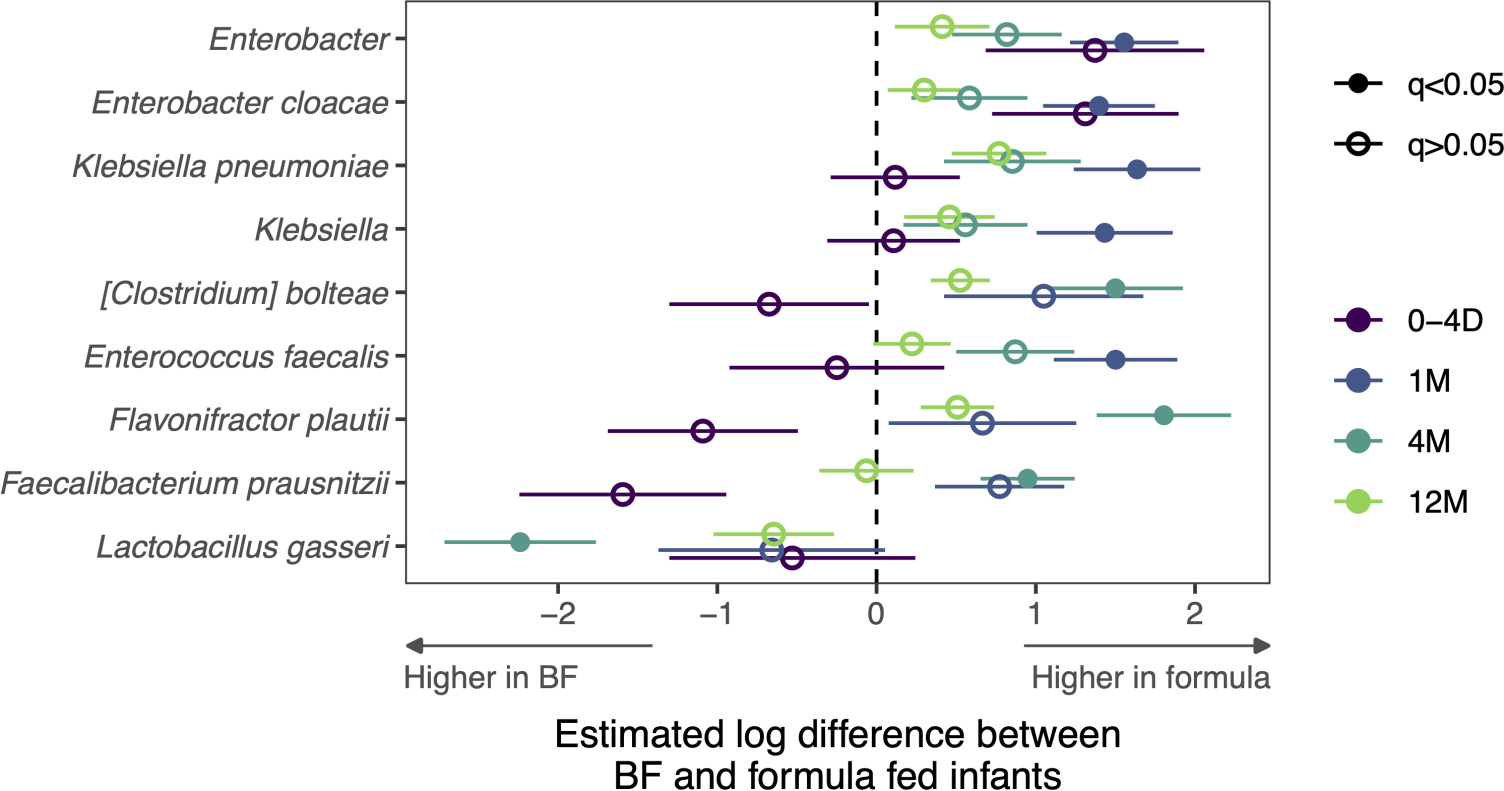
Bacterial community composition is correlated with feeding type during early life. Metagenomic analyses displaying enriched bacteria with mean relative abundance >0.5% across all samples in formula versus breastfed infants (*n* = 17 for breastfed and *n* = 13 for formula-fed). Linear mixed-effects modeling was used with fdr <0.05 considered statistically significant.

**Fig 7:**
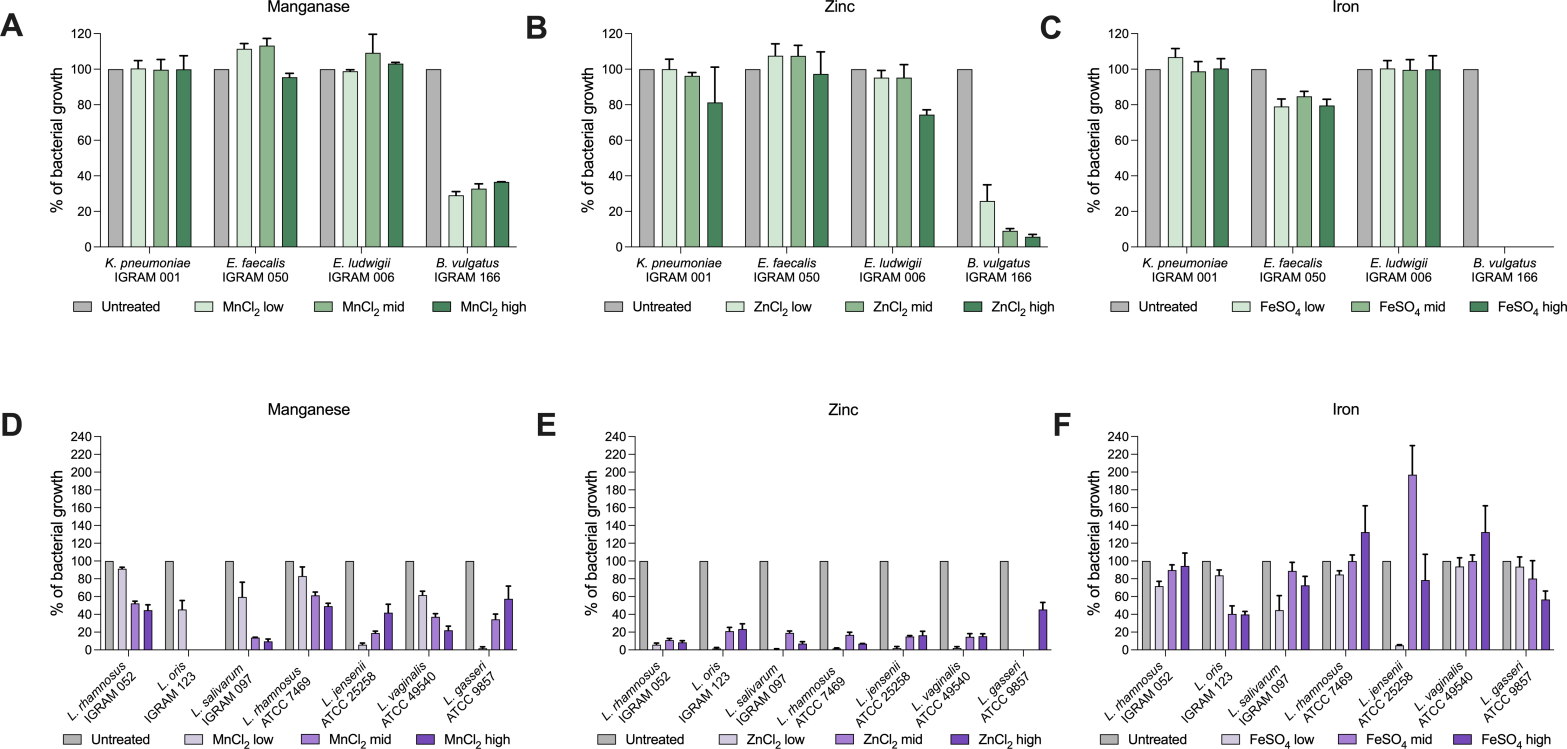
Excess metal levels differentially impact growth of early life colonizers. *In vitro* growth at 10 h of *Klebsiella*, *Enterococcus*, *Enterobacter*, and *Bacteroides* strains in untreated BHIS/MRSB or with: (**A**) 1.25 mM of MnCl_2_ (MnCl_2_ low), 2.5 mM of MnCl_2_ (MnCl_2_ mid) or 5 mM of MnCl_2_ (MnCl_2_ high); (**B**) 1.25 mM of ZnCl_2_ (ZnCl_2_ low), 2.5 mM of ZnCl_2_ (ZnCl_2_ mid) or 5 mM of ZnCl_2_ (ZnCl_2_ high); and (**C**) 1.25 mM of FeSO_4_ (FeSO_4_ low), 2.5 mM of FeSO_4_ (FeSO_4_ mid) or 5 mM of FeSO_4_ (FeSO_4_ high). *In vitro* growth at 10 h of *Lactobacillus* strains in untreated BHIS/MRSB or with: (**D**) 1.25 mM of MnCl_2_ (MnCl_2_ low), 2.5 mM of MnCl_2_ (MnCl_2_ mid) or 5 mM of MnCl_2_ (MnCl_2_ high); (**E**) 1.25 mM of ZnCl_2_ (ZnCl_2_ low), 2.5 mM of ZnCl_2_ (ZnCl_2_ mid) or 5 mM of ZnCl_2_ (ZnCl_2_ high); and (**F**) 1.25 mM of FeSO_4_ (FeSO_4_ low), 2.5 mM of FeSO_4_ (FeSO_4_ mid) or 5 mM of FeSO_4_ (FeSO_4_ high) (Error bars indicate mean with SD).

Like metal restriction, excess metals can impact the ecology of the gut by altering nutritional niches and causing toxicity to bacterial cells (23). We hypothesized that high levels of metals may impact the gut microbiota in formula-fed infants. To test this, we examined the microbial communities of breast and formula-fed infants in sub-study 2 using differential abundance analyses. We observed formula-fed infants harbored higher abundances of some *Enterococcus*, *Klebsiella*, *Enterobacter*, and *Clostridia*; while *L. gasseri* was higher in breastfed infants at 4 months (Fig. 6). Based on this, we postulated that microbiota enriched in formula-fed infants may be more resistant to the toxic effects of metals. Thus, we measured the growth of bacterial isolates from these infants in highly concentrated metal environments to model the potential toxic effect of metals in these conditions. We observed that IGraM isolates from the genera *Klebsiella*, *Enterobacter*, and *Enterococcus* were resistant to high concentrations of zinc, manganese, and iron (Fig. 7A through C). However, bacteria from the genus *Bacteroides* were susceptible to toxicity from high concentrations of these metals (Fig. 7A through C). *Lactobacillus* strains were sensitive to high concentrations of manganese (Fig. 7D) and hypersensitive to zinc (Fig. 7E). Interestingly, we observed the converse relationship with iron, as increasing concentrations enhanced growth (Fig. 7F). One exception was a strain of *L. oris* that was isolated from the stool of an infant from the IGraM cohort, which was sensitive to high levels of iron. Despite *Lactobacillus* having low iron demands, enhanced growth with iron supplementation is consistent with previous reports (36). These data collectively suggest that dietary metals may play a critical role in shaping the fitness of early life colonizing microbiota in the first year of life. Taken together, this study suggests that host and dietary factors may reshape metal availability in the gut during the first year of life, which could play a central role in the rules that govern gut microbiota colonization and succession.

## DISCUSSION

The gut microbiota plays a critical role in host development and health. Understanding how this microbial community is established early in life is essential to our understanding of long-term health and will support the creation of the next generation of microbial therapeutics. In this study, we provide evidence that nutritional immunity and dietary metals may shape the microbial community composition of infants. Specifically, we show that high levels of calprotectin in the infant gut are associated with altered microbial composition and abundance. We further demonstrate that infant strains of *Lactobacillus* are sensitive to metal restriction, whereas bacteria from the *Enterobacteriaceae* family and *Enterococcus* and *Bacteroides* genera thrive in metal-depleted environments. These data highlight the potentially important role that host-mediated nutritional immunity may have on the gut ecosystem and microbiota during early life and highlight a novel mechanism for the restriction of certain microbiota, like the Lactobacilli, at this early time point.

Dietary metals play an important role in host health, while also shaping the gut microbiota and outcomes of infection (21, 23). Moreover, feeding practices have a strong effect on the microbial composition in the infant gut. Specifically, children who are primarily formula-fed have distinct, more mature microbiomes compared to infants who are breastfed. However, little is known about the role of dietary metals on the early life microbiota and the nutritional factors in breast milk and formula that drive these distinct microbial profiles. We show that formula-fed infants harbor higher levels of metals in their GI tract. We also show that high levels of metals correlate with higher abundances of bacteria from the genus *Klebsiella*, *Enterococcus*, and *Enterobacter* and negatively correlate with members of the *Bacteroides* genus. We further demonstrate that high levels of metals do not have a toxic effect on the growth of infant isolates from the genera *Klebsiella*, *Enterococcus*, and *Enterobacter*, whereas the *Bacteroides* and *Lactobacillus* grow poorly in these conditions. These data demonstrate a possible mechanism by which feeding practices shape microbial composition and assembly during early life.

The findings reported in this study serve as an early foundation for future studies into the role of nutritional immunity during early life. Nutritional immunity has been deeply explored in the context of host-pathogen interactions in numerous infections and tissues (18, 20, 22). However, little is known about the impact of nutritional immunity in the gut and nutritional immunity in the dynamic early life gut ecosystem has gone largely unexplored (23). Our study is limited in scope, as we are unable to directly test the effect of calprotectin on the gut environment of humans. Moreover, our work does not explore the potential role of other host factors involved in nutritional immunity, including lactoferrin. Future studies involving complex models of community succession will shed light on the impact of both metal limitation and metal supplementation on shaping community assembly and fitness of individual early colonizers. Importantly, the modest number of human infant samples in our study limits our power to generate significant correlations between calprotectin levels and the relative abundance of early life colonizers. Thus, future studies with larger cohorts are needed to understand the effects of metal limitation in the assembly of the early life microbiota in humans. The findings that calprotectin levels at birth are negatively correlated with gestational age and not correlated with markers of intestinal inflammation at 1 month, suggest that the role of calprotectin early in life is different than its role later in life. Future studies are warranted to determine the physiologic role of calprotectin in the development of the infant GI tract. Despite these limitations, this study describes for the first time the importance of the metal paradox in early life microbial colonization and succession (22). Specifically, bacteria likely need to simultaneously manage metal-deplete and metal-replete conditions to thrive in the early life gut. To survive in excess metal environments, bacteria must employ strategies to detoxify and export metals. Conversely, in metal-depleted environments driven by host-mediated nutritional immunity and microbe-microbe competition, bacteria must scavenge and import metals to meet their nutritional needs (37). This study suggests that the ability to perform either or both of these functions is key to surviving and persisting in the volatile and dynamic early life environment. In conclusion, this work highlights an unappreciated observation that metals may be a key nutrient that shapes the composition of the gut microbiota early in life. Our findings provide new insights into the biological processes that govern early life community assembly involving the impact of feeding methods early in life and the role of host-factors influencing the microbiota.

## MATERIALS AND METHODS

### Human subjects

Study participants were enrolled in the IGraM Study, a prospective, longitudinal cohort study of pregnant African American women and their infants (24). The study protocol was reviewed and approved by the Committee for the Protection of Human Subjects (Internal Review Board) of the Children’s Hospital of Philadelphia, with number 14-010833. Data used in this publication were consistent with the stated purpose of the research. The Institutional Review Board-approved consent documents included language that allowed participants to indicate whether they would like to have their information included in future research. Subjects may participate in the original research without their information (even if de-identified) being included in future research. Two related sub-studies from the larger IGraM cohort were used in our study. Sub-study 1 collected samples from 17 infants weekly from birth (week 0) through 1 month of age (week 4), and then 1 month, 4 months, and 1 year of age. Sub-study 2 collected samples from 30 infants at birth, 1 month, 4 months, and 1 year of age.

### Bacterial strains and isolation from human fecal samples

All bacterial strains used in this study and their origin are shown in Table S1. Strains were either acquired from ATCC or isolated from human infant stool samples from this study. For bacterial strains isolated from fecal samples, 50–100 mg of stool was inoculated in warm thioglycolate broth and incubated at 37°C aerobically. After 24 h, 10 mL of the broth was plated onto tryptic soy agar (BD) with sheep blood (SBA), MacConkey agar (BD), Columbia agar, Yeast Casitone Fatty Acids Agar with Carbohydrates (YCFAC) (Anaerobe Systems), and De Man, Rogosa and Sharpe agar (MRSA) (Millipore Sigma) and incubated aerobically and anaerobically at 37°C. After 24 h, colonies were morphologically characterized and inoculated in either Brain Heart Infusion (BD) supplemented with yeast extract (BD) (BHIS) or De Man, Rogosa and Sharpe broth (MRSB) and incubated at 37°C. All strains were frozen in glycerol stocks at −80°C. For DNA extraction and identification, 1 mL of the culture was pelleted by centrifugation at 4,000 × *g* for 10 min. Supernatants were discarded and bacterial pellets were resuspended in phosphate-buffered saline (PBS) and transferred to a 2 mL 0.1 mm glass bead screw cap tube (Qiagen). Cultures were shaken for 1 min at 2,000 rpm using a PowerLyzer Homogenizer (Qiagen). Lysed samples were centrifuged at 8,000 rpm for 2 min and 180 mL of supernatant was transferred into a microcentrifuge tube and DNA was extracted using the DNeasy Blood and Tissue Kit (Qiagen) with a QIAcube (QIAGEN) following the manufacturer’s protocol.

After DNA was extracted, 16S rRNA gene was amplified via PCR using the Phusion High-Fidelity Polymerase (Thermo Fisher Scientific) following the manufacturer’s instruction and using the following 16S rRNA DNA forward and reverse primers f: AGRGTTTGATYMTGGCTCAG and r: GGYTACCTTGTTACGACT. 16 rRNA gene amplification was confirmed through agarose gel electrophoresis and the amplicon product was subsequently cleaned using Monarch PCR and DNA CleanUp Kit (New England Biolabs) following the manufacturer’s instructions. About 150 ng of the clean DNA samples was submitted for Sanger sequencing at the Children’s Hospital of Philadelphia sequencing core.

### Bacterial growth curves

All bacterial growth was performed in anaerobic conditions in an anaerobic chamber (90% nitrogen, 5% hydrogen, 5% carbon dioxide, Coy Lab Products) to best model growth in the gastrointestinal tract. Bacterial colonies were inoculated in a mixture of 4:1 BHIS/MRSB and grown for 16 h overnight. The next day, cultures were normalized to optical density at 600 nm (OD_600_) of 0.5. Bacteria were grown at a 1:50 dilution in a 96-well plate with increasing concentrations of TPEN, manganese chloride tetrahydrate (MnCl_2_) (Fisher Scientific), zinc chloride (ZnCl_2_) (Alfa Aesar), or iron(III) sulfate heptahydrate (FeSO_4_) (Acro Organics) at a final volume of 200 µL. Bacteria were grown with double orbital continuous shaking for 16–24 h at 37°C in a Synergy HTX Multi-Mode Reader (Biotek) plate reader measuring OD_600_ every 20 min.

### Shotgun metagenomic DNA sequencing

DNA was extracted from fecal samples using the PowerSoil-htp kit (MO BIO Laboratories, Carlsbad, CA), following the manufacturer’s instructions, with the optional heating step included (MO BIO has since been purchased by QIAGEN; the extraction kit is now sold as the DNeasy PowerSoil HTP 96 Kit). Negative control extraction samples were included in parallel to all fecal sample extractions. Shotgun libraries were generated from 1 ng of DNA using the NexteraXT kit (Illumina, San Diego, CA, USA). Libraries were sequenced on the Illumina HiSeq using 2 × 125 bp chemistry in High Output mode.

Fifteen negative control samples were included: one sample of unsoiled diaper (diaper blank), five unused swab tip samples (blank swabs), and nine samples of DNA-free water added to the NexteraXT library preparation kit instead of DNA (library negative controls). Negative controls for the sequencing kit without DNA-free water were not included.

Paired-end reads from metagenomics shotgun sequencing were processed using the Sunbeam pipeline v1.0.0 (38). Sequence reads were quality-filtered and Illumina adapter sequences were removed using Trimmomatic v0.33 (39). Low-complexity reads that fell below the default threshold were marked and removed using Komplexity v0.3.0 (38). Reads that aligned to the human genome (hg38) or to the genome of phage phiX (which is used in sequencing library prep) using BWA v0.7.3 (40) were removed. With the remaining read pairs, we carried out taxonomic classification using MetaPhlAn v2.0 (MetaPhlAn2) (41).

### Calprotectin quantification

Approximately 20 mg of stool sample was diluted in 1 mL of PBS. Samples were homogenized using a sterile wooden stick and shaken vigorously for 30 s by vortexing. Next, samples were centrifuged for 20 min at 10,000 × *g*. Supernatants were diluted to 1:400 and levels of calprotectin were measured using the QUANTA Lite Calprotectin Extended Range (Werfen) following the manufacturer’s instructions.

### Metal measurements in stool

Fecal material was transferred to tared 15 mL conical tubes and weighed to determine the amount of feces used in the analysis. 2.5, 1.25, 0.625, or 0.25 mL of 4:1 OPTIMA grade nitric acid:ultratrace hydrogen peroxide was added to each sample, depending on the fecal material weight. Samples were incubated for 48 h at 65°C to completely digest the fecal material. Following digestion, 12.5, 7, 4, or 2 mL of Ultrapure water was added to each sample to dilute the nitric acid concentration to less than 10%.

Elemental quantification on acid-digested stool samples was performed using an Agilent 7700 inductively coupled plasma mass spectrometer (Agilent, Santa Clara, CA) attached to a Teledyne CETAC Technologies ASX-560 autosampler (Teledyne CETAC Technologies, Omaha, NE). The following settings were fixed for the analysis: Cell Entrance = −40 V; Cell Exit = −60 V; Plate Bias = −60 V; OctP Bias = −18 V; and collision cell Helium Flow = 4.5  mL/min. Optimal voltages for Extract 2, Omega Bias, Omega Lens, OctP RF, and Deflect were determined empirically before each sample set was analyzed. Element calibration curves were generated using ARISTAR ICP Standard Mix (VWR, Radnor, PA). Samples were introduced by peristaltic pump with 0.5  mm internal diameter tubing through a MicroMist borosilicate glass nebulizer (Agilent). Samples were initially taken up at 0.5 rps for 30 s followed by 30 s at 0.1 rps to stabilize the signal. Samples were analyzed in Spectrum mode at 0.1 rps, collecting three points across each peak and performing three replicates of 100 sweeps for each element analyzed. The sampling probe and tubing were rinsed for 20 s at 0.5 rps with 2% nitric acid between every sample. Data were acquired and analyzed using the Agilent Mass Hunter Workstation Software version A.01.02. Data of the investigated metal ions were normalized to the fecal material weight of each sample.

### Statistical analysis

Analyses were conducted in GraphPad Prism 10 (Boston, MA) or R (42) using linear mixed-effects modeling or repeated measured ANOVA, depending on the presence of mixing values. Individual comparisons were conducted using Tukey’s or Dunnett’s multiple comparisons tests, or paired/un-paired *t* tests with false discovery rate correction for multiple corrections (43).

## Data Availability

The IGraM study enrolled pregnant African American mothers and their newborn infants. The broad purpose of the research study was to learn more about the bacteria normally living in the child’s gut, how it is transferred from mother to child and whether it affects the child’s growth in the first three years of life. The Institutional Review Board-approved consent documents included language that allowed participants to indicate whether they would like to have their information included in future research. Subjects may participate in the original research without their information (even if de-identified) being included in future research. Therefore, the data submitted to the repository (SRA accessions PRJNA1145027, PRJNA1106565, and PRJNA1042647) were limited to those individuals who consented to future use of their data and are not the entire data set used in the analyses presented here. To request the complete data set, authors can be contacted with a summary of how the data will be used and how it is consistent with the goals of the approved study.

## SUPPLEMENTAL MATERIAL

Supplemental Material mbio.01534-24-s0001.docx Supplemental figures and table.

ASM does not own the copyrights to Supplemental Material that may be linked to, or accessed through, an article. The authors have granted ASM a non-exclusive, world-wide license to publish the Supplemental Material files. Please contact the corresponding author directly for reuse.

## References

[1] Savage DC. 1977. Microbial ecology of the gastrointestinal tract. Annu Rev Microbiol 31:107–133. doi:10.1146/annurev.mi.31.100177.000543 334036

[2] Gensollen T, Iyer SS, Kasper DL, Blumberg RS. 2016. How colonization by microbiota in early life shapes the immune system. Science 352:539–544. doi:10.1126/science.aad9378 27126036 PMC5050524

[3] Al Nabhani Z, Dulauroy S, Marques R, Cousu C, Al Bounny S, Déjardin F, Sparwasser T, Bérard M, Cerf-Bensussan N, Eberl G. 2019. A weaning reaction to microbiota is required for resistance to immunopathologies in the adult. Immunity 50:1276–1288. doi:10.1016/j.immuni.2019.02.014 30902637

[4] Gollwitzer ES, Marsland BJ. 2015. Impact of early-life exposures on immune maturation and susceptibility to disease. Trends Immunol 36:684–696. doi:10.1016/j.it.2015.09.009 26497259

[5] Borbet TC, Pawline MB, Zhang X, Wipperman MF, Reuter S, Maher T, Li J, Iizumi T, Gao Z, Daniele M, Taube C, Koralov SB, Müller A, Blaser MJ. 2022. Influence of the early-life gut microbiota on the immune responses to an inhaled allergen. Mucosal Immunol 15:1000–1011. doi:10.1038/s41385-022-00544-5 35842561 PMC9835105

[6] Abrahamsson TR, Jakobsson HE, Andersson AF, Björkstén B, Engstrand L, Jenmalm MC. 2012. Low diversity of the gut microbiota in infants with atopic eczema. J Allergy Clin Immunol 129:434–440, doi:10.1016/j.jaci.2011.10.025 22153774

[7] Zhang C, Li L, Jin B, Xu X, Zuo X, Li Y, Li Z. 2021. The effects of delivery mode on the gut microbiota and health: state of art. Front Microbiol 12:724449. doi:10.3389/fmicb.2021.724449 35002992 PMC8733716

[8] Reyman M, van Houten MA, van Baarle D, Bosch AATM, Man WH, Chu MLJN, Arp K, Watson RL, Sanders EAM, Fuentes S, Bogaert D. 2019. Impact of delivery mode-associated gut microbiota dynamics on health in the first year of life. Nat Commun 10:4997. doi:10.1038/s41467-019-13014-7 31676793 PMC6825150

[9] Arrieta MC, Stiemsma LT, Amenyogbe N, Brown EM, Finlay B. 2014. The intestinal microbiome in early life: health and disease. Front Immunol 5:427. doi:10.3389/fimmu.2014.00427 25250028 PMC4155789

[10] Matamoros S, Gras-Leguen C, Le Vacon F, Potel G, de La Cochetiere M-F. 2013. Development of intestinal microbiota in infants and its impact on health. Trends Microbiol 21:167–173. doi:10.1016/j.tim.2012.12.001 23332725

[11] Gajer P, Brotman RM, Bai G, Sakamoto J, Schütte UME, Zhong X, Koenig SSK, Fu L, Ma ZS, Zhou X, Abdo Z, Forney LJ, Ravel J. 2012. Temporal dynamics of the human vaginal microbiota. Sci Transl Med 4:132ra152. doi:10.1126/scitranslmed.3003605 PMC3722878 22553250

[12] Bezirtzoglou E, Tsiotsias A, Welling GW. 2011. Microbiota profile in feces of breast- and formula-fed newborns by using fluorescence in situ hybridization (FISH). Anaerobe 17:478–482. doi:10.1016/j.anaerobe.2011.03.009 21497661

[13] Ma J, Li Z, Zhang W, Zhang C, Zhang Y, Mei H, Zhuo N, Wang H, Wang L, Wu D. 2020. Comparison of gut microbiota in exclusively breast-fed and formula-fed babies: a study of 91 term infants. Sci Rep 10:15792. doi:10.1038/s41598-020-72635-x 32978424 PMC7519658

[14] German JB, Freeman SL, Lebrilla CB, Mills DA. 2008. Human milk oligosaccharides: evolution, structures and bioselectivity as substrates for intestinal bacteria. Nestle Nutr Workshop Ser Pediatr Program 62:205–218; doi:10.1159/000146322 PMC2861563 18626202

[15] Guo J, Ren C, Han X, Huang W, You Y, Zhan J. 2021. Role of IgA in the early-life establishment of the gut microbiota and immunity: Implications for constructing a healthy start. Gut Microbes 13:1–21. doi:10.1080/19490976.2021.1908101 PMC8078773 33870860

[16] Trend S, Strunk T, Hibbert J, Kok CH, Zhang G, Doherty DA, Richmond P, Burgner D, Simmer K, Davidson DJ, Currie AJ. 2015. Antimicrobial protein and peptide concentrations and activity in human breast milk consumed by preterm infants at risk of late-onset neonatal sepsis. PLoS One 10:e0117038. doi:10.1371/journal.pone.0117038 25643281 PMC4314069

[17] McKeen S, Roy NC, Mullaney JA, Eriksen H, Lovell A, Kussman M, Young W, Fraser K, Wall CR, McNabb WC. 2022. Adaptation of the infant gut microbiome during the complementary feeding transition. PLoS One 17:e0270213. doi:10.1371/journal.pone.0270213 35834499 PMC9282554

[18] Monteith AJ, Skaar EP. 2021. The impact of metal availability on immune function during infection. Trends Endocrinol Metab 32:916–928. doi:10.1016/j.tem.2021.08.004 34483037 PMC8516721

[19] Andreini C, Bertini I, Cavallaro G, Holliday GL, Thornton JM. 2008. Metal ions in biological catalysis: from enzyme databases to general principles. J Biol Inorg Chem 13:1205–1218. doi:10.1007/s00775-008-0404-5 18604568

[20] Becker KW, Skaar EP. 2014. Metal limitation and toxicity at the interface between host and pathogen. FEMS Microbiol Rev 38:1235–1249. doi:10.1111/1574-6976.12087 25211180 PMC4227937

[21] Zackular J. P., Chazin WJ, Skaar EP. 2015. Nutritional immunity: S100 proteins at the host-pathogen interface. J Biol Chem 290:18991–18998. doi:10.1074/jbc.R115.645085 26055713 PMC4521021

[22] Murdoch CC, Skaar EP. 2022. Nutritional immunity: the battle for nutrient metals at the host-pathogen interface. Nat Rev Microbiol 20:657–670. doi:10.1038/s41579-022-00745-6 35641670 PMC9153222

[23] Zackular JP, Moore JL, Jordan AT, Juttukonda LJ, Noto MJ, Nicholson MR, Crews JD, Semler MW, Zhang Y, Ware LB, Washington MK, Chazin WJ, Caprioli RM, Skaar EP. 2016. Dietary zinc alters the microbiota and decreases resistance to Clostridium difficile infection. Nat Med 22:1330–1334. doi:10.1038/nm.4174 27668938 PMC5101143

[24] Bittinger K, Zhao C, Li Y, Ford E, Friedman ES, Ni J, Kulkarni CV, Cai J, Tian Y, Liu Q, et al.. 2020. Bacterial colonization reprograms the neonatal gut metabolome. Nat Microbiol 5:838–847. doi:10.1038/s41564-020-0694-0 32284564 PMC8052915

[25] Willers M, Ulas T, Völlger L, Vogl T, Heinemann AS, Pirr S, Pagel J, Fehlhaber B, Halle O, Schöning J, et al.. 2020. S100A8 and S100A9 are important for postnatal development of gut microbiota and immune system in mice and infants. Gastroenterology 159:2130–2145. doi:10.1053/j.gastro.2020.08.019 32805279

[26] Korndörfer IP, Brueckner F, Skerra A. 2007. The crystal structure of the human (S100A8/S100A9)2 heterotetramer, calprotectin, illustrates how conformational changes of interacting alpha-helices can determine specific association of two EF-hand proteins. J Mol Biol 370:887–898. doi:10.1016/j.jmb.2007.04.065 17553524

[27] Jukic A, Bakiri L, Wagner EF, Tilg H, Adolph TE. 2021. Calprotectin: from biomarker to biological function. Gut 70:1978–1988. doi:10.1136/gutjnl-2021-324855 34145045 PMC8458070

[28] Bjarnason I. 2017. The use of fecal calprotectin in inflammatory bowel disease. Gastroenterol Hepatol (N Y) 13:53–56. 28420947 PMC5390326

[29] Sipponen T, Kolho KL. 2015. Fecal calprotectin in diagnosis and clinical assessment of inflammatory bowel disease. Scand J Gastroenterol 50:74–80. doi:10.3109/00365521.2014.987809 25523558

[30] Li F, Ma J, Geng S, Wang J, Liu J, Zhang J, Sheng X. 2015. Fecal calprotectin concentrations in healthy children aged 1-18 months. PLoS One 10:e0119574. doi:10.1371/journal.pone.0119574 25742018 PMC4351193

[31] Günaydın Şahin BS, Keskindemirci G, Özden TA, Durmaz Ö, Gökçay G. 2020. Faecal calprotectin levels during the first year of life in healthy children. J Paediatrics Child Health 56:1806–1811. doi:10.1111/jpc.14933 32502317

[32] Olafsdottir E, Aksnes L, Fluge G, Berstad A. 2002. Faecal calprotectin levels in infants with infantile colic, healthy infants, children with inflammatory bowel disease, children with recurrent abdominal pain and healthy children. Acta Paediatr 91:45–50. doi:10.1080/080352502753457932 11883817

[33] Lee YM, Min C-Y, Choi YJ, Jeong SJ. 2017. Delivery and feeding mode affects fecal calprotectin levels in infants <7months old. Early Hum Dev 108:45–48. doi:10.1016/j.earlhumdev.2017.03.014 28391117

[34] Lee J-Y, Cevallos SA, Byndloss MX, Tiffany CR, Olsan EE, Butler BP, Young BM, Rogers AWL, Nguyen H, Kim K, Choi S-W, Bae E, Lee JH, Min U-G, Lee D-C, Bäumler AJ. 2020. High-fat diet and antibiotics cooperatively impair mitochondrial bioenergetics to trigger dysbiosis that exacerbates pre-inflammatory bowel disease. Cell Host Microbe 28:273–284. doi:10.1016/j.chom.2020.06.001 32668218 PMC7429289

[35] Lopez CA, Beavers WN, Weiss A, Knippel RJ, Zackular JP, Chazin W, Skaar EP. 2019. The immune protein calprotectin impacts clostridioides difficile metabolism through zinc limitation. MBio 10:e02289-19. doi:10.1128/mBio.02289-19 31744916 PMC6867894

[36] Huynh U, Qiao M, King J, Trinh B, Valdez J, Haq M, Zastrow ML. 2022. Differential effects of transition metals on growth and metal uptake for two distinct Lactobacillus species. Microbiol Spectr 10:e0100621. doi:10.1128/spectrum.01006-21 35080431 PMC8791193

[37] Pajarillo EAB, Lee E, Kang DK. 2021. Trace metals and animal health: interplay of the gut microbiota with iron, manganese, zinc, and copper. Anim Nutr 7:750–761. doi:10.1016/j.aninu.2021.03.005 34466679 PMC8379138

[38] Clarke EL, Taylor LJ, Zhao C, Connell A, Lee J-J, Fett B, Bushman FD, Bittinger K. 2019. Sunbeam: an extensible pipeline for analyzing metagenomic sequencing experiments. Microbiome 7:46. doi:10.1186/s40168-019-0658-x 30902113 PMC6429786

[39] Bolger AM, Lohse M, Usadel B. 2014. Trimmomatic: a flexible trimmer for Illumina sequence data. Bioinformatics 30:2114–2120. doi:10.1093/bioinformatics/btu170 24695404 PMC4103590

[40] Li H, Durbin R. 2009. Fast and accurate short read alignment with Burrows-Wheeler transform. Bioinformatics 25:1754–1760. doi:10.1093/bioinformatics/btp324 19451168 PMC2705234

[41] Truong DT, Franzosa EA, Tickle TL, Scholz M, Weingart G, Pasolli E, Tett A, Huttenhower C, Segata N. 2015. MetaPhlAn2 for enhanced metagenomic taxonomic profiling. Nat Methods 12:902–903. doi:10.1038/nmeth.3589 26418763

[42] R. C. Team. 2013. R: a language and environment for statistical computing. Vienna, Austria. Available from: http://www.R-project.org

[43] Benjamini Y, Hochberg Y. 1995. Controlling the false discovery rate: a practical and powerful approach to multiple testing. J R Stat Soc Ser B 57:289–300. doi:10.1111/j.2517-6161.1995.tb02031.x

