# Supplementary material for: Metal availability shapes early life microbial ecology and community succession
Source: mBio. 2024 Oct 23;15(11):e01534-24. doi: 10.1128/mbio.01534-24 (PMC11558993; doi:10.1128/mbio.01534-24)
Supplement: Supplemental Material — Supplemental figures and table. [file mbio.01534-24-s0001.docx]

Supplementary Materials for

**Metal availability shapes early life microbial ecology and community succession**

Joshua Soto Ocaña^1,2#^, Elliot S. Friedman^3#^, Orlaith Keenan^1,2^, Nile U. Bayard^1^, Eileen Ford^4,5^, Ceylan Tanes^4^, Matthew J. Munneke^6^, William N. Beavers^6^, Eric P. Skaar^6^, Kyle Bittinger^4,7^, Babette S. Zemel^4,5^, Gary D. Wu^3^*, Joseph P. Zackular^1,2,7,^*

Corresponding authors:

Gary D. Wu: gdwu@pennmedicine.upenn.edu

Joseph P. Zackular: joseph.zackular@pennmedicine.upenn.edu

**SUPPLEMENTARY FIGURES**


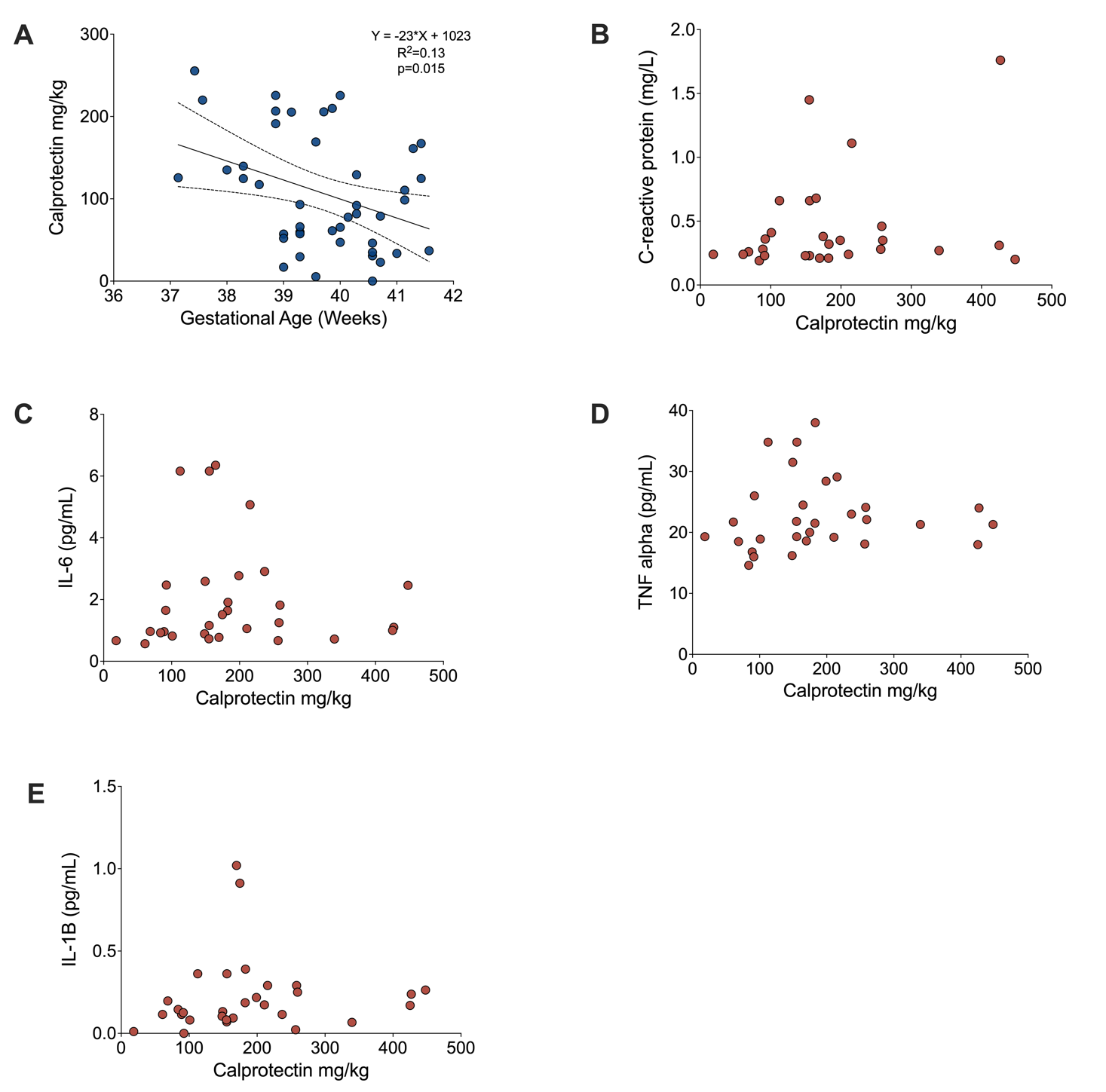


**Supplementary Figure 1. Calprotectin levels at birth are negatively correlated with gestational age, but are not correlated with markers of intestinal inflammation at one month**

(A) Simple linear regression of gestational age (weeks) with fecal calprotectin (mg/kg) at birth (n=44; p=0.015). Simple linear regression of fecal calprotectin (mg/kg) with (B-E) C-reactive protein, IL-6, TNF alpha, and IL-1B at one month (n=28 for C-reactive protein and n=30 for IL-6, TNF alpha, and IL-1B; p > 0.05).


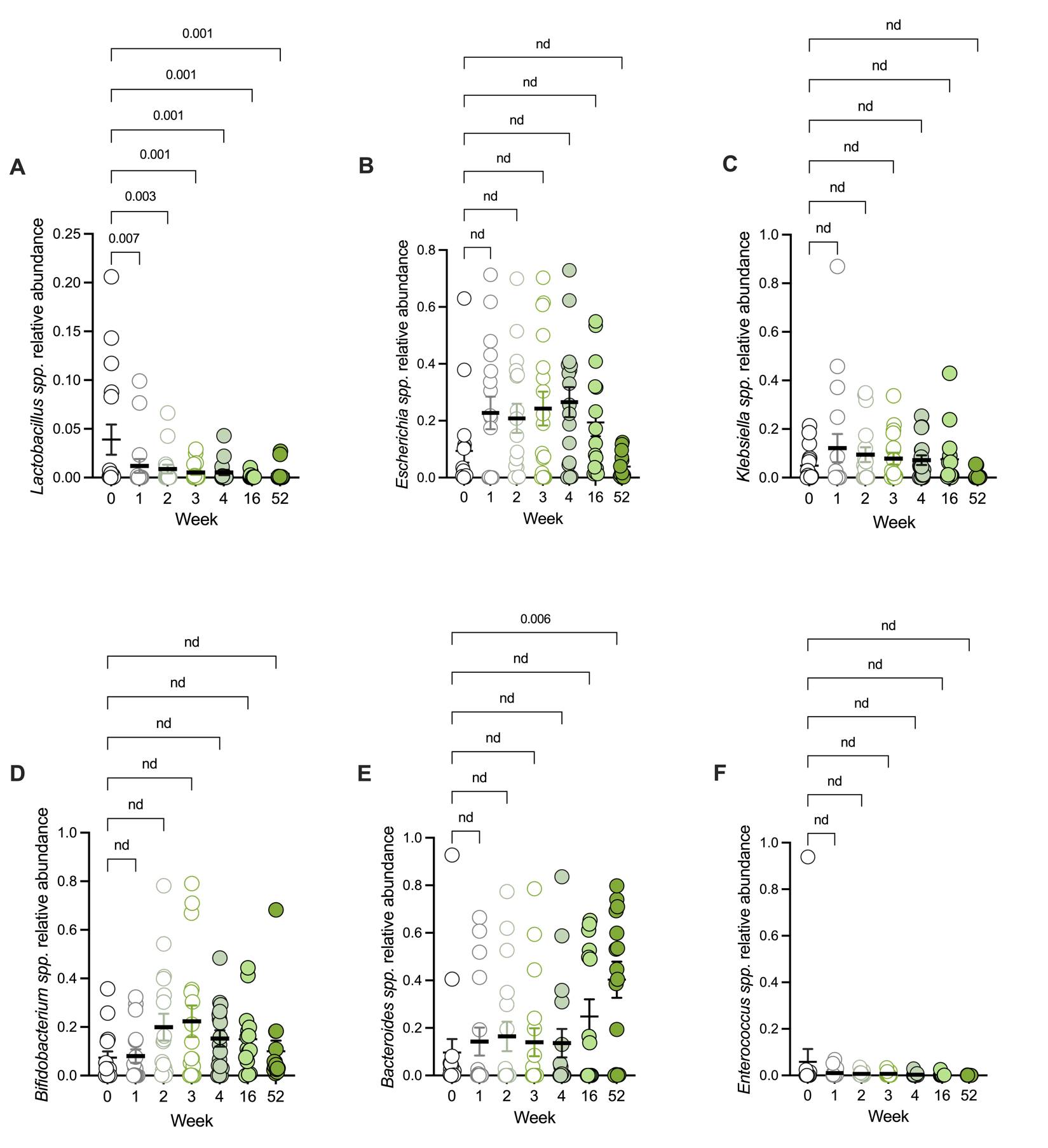


**Supplementary Figure 2. *Lactobacillus* abundance decreases following birth.**

(A-F) Metagenomic analyses of *Lactobacillus, Escherichia, Klebsiella, Bacteroides, Bifidobacterium,* and *Enterococcus* relative abundances in infant human stool samples (n=17, paired t-tests with false discovery rate corrections for multiple comparisons, *fdr < 0.05).


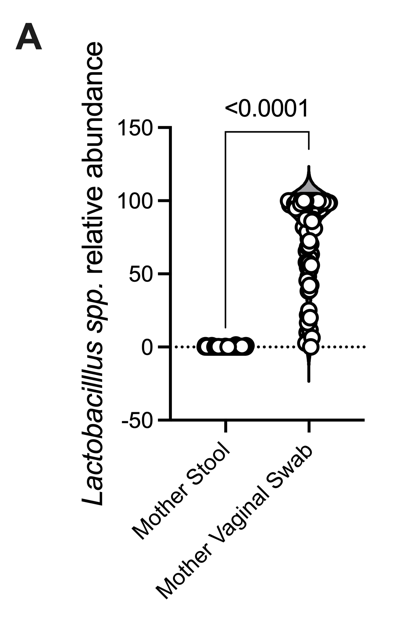


**Supplementary Figure 3. Mother’s vaginal microbiomes are enriched with *Lactobacillus* species**

Metagenomic analyses of *Lactobacillus* relative abundances of IGraM infant’s mothers in stool samples and vaginal swab at birth.


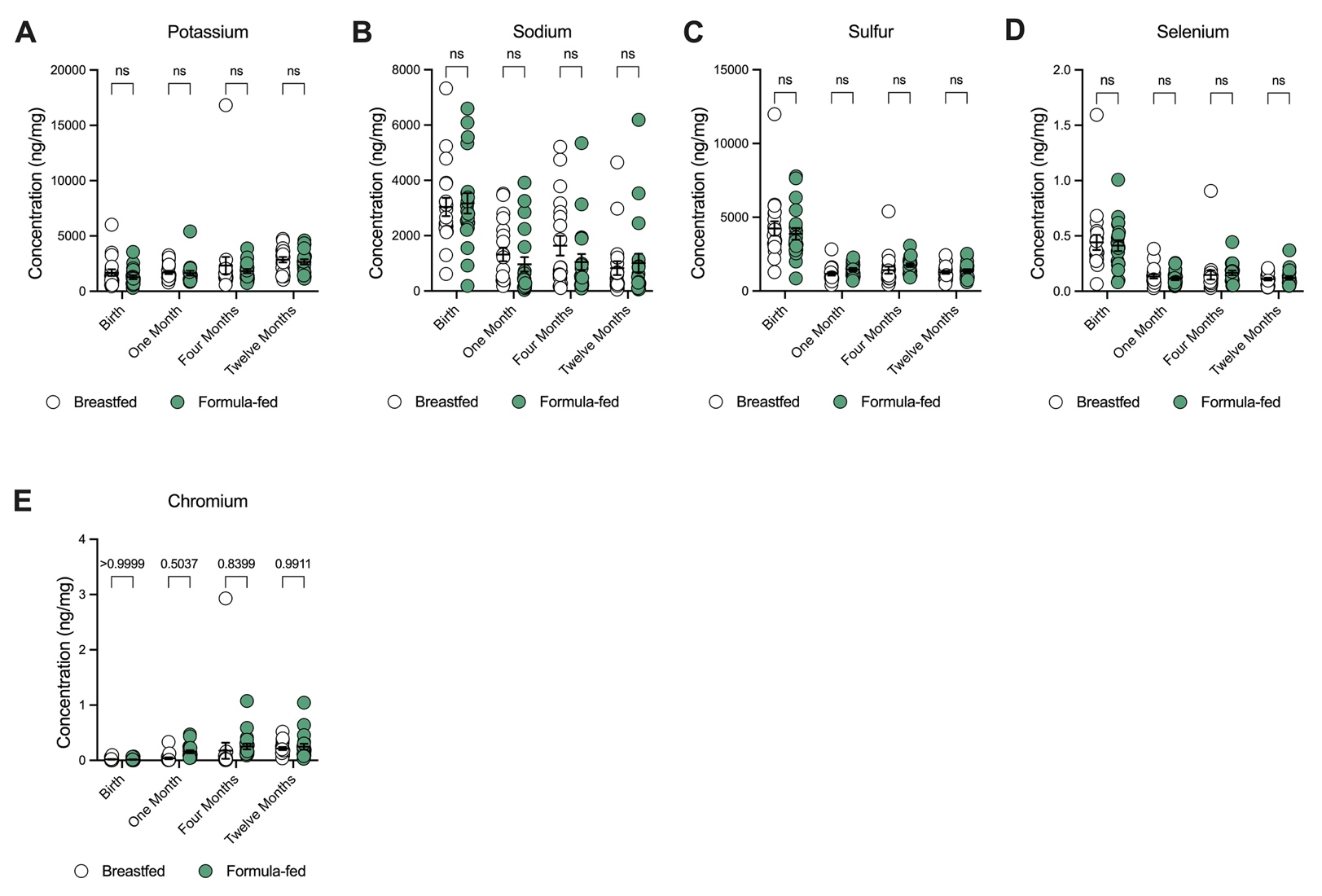


**Supplementary Figure 4. Potassium, sodium, sulfur, selenium, and chromium are not affected by formula feeding in infants**

(A-E) Elemental metals quantification in human infant stool samples. ANOVA with Geisser-Greenhouse correction was performed to assess statistical significance.

**SUPPLEMENTARY TABLES**

**Supplementary Table 1. Bacterial strains**

| **Species** | **Strain** | **Growth on agar** |
| --- | --- | --- |
| *Enterococcus faecalis* | OG1RF | BHISA |
| *Enterococcus faecalis* | IGraM 050 | BHISA |
| *Enterococcus faecium* | ATCC 700221 | BHISA |
| *Enterococcus faecium* | IGraM 033 | BHISA |
| *Enterococcus gallinarium* | IGraM 067 | BHISA |
| *Enterococcus hirae* | IGraM 083 | BHISA |
| *Enterococcus avium* | IGraM 135 | BHISA |
| *Escherichia coli* | DH5α | BHISA |
| *Escherichia coli* | MG 1655 | BHISA |
| *Escherichia coli* | IGraM 002 | BHISA |
| *Klebsiella pneumoniae* | IGraM 001 | BHISA |
| *Enterobacter ludwigii* | IGraM 006 | BHISA |
| *Proteus mirabalis* | IGraM 027 | BHISA |
| *Shigella flexneri* | IGraM 040 | BHISA |
| *Bacteroides vulgatus* | ATCC 8482 | BHISA |
| *Bacteroides vulgatus* | IGraM 166 | BHISA |
| *Bacteroides dentium* | IGraM 059 | BHISA |
| *Bacteroides ovatus* | IGraM 167 | BHISA |
| *Lactobacillus rhamnosus* | ATCC 7469 | MRSA |
| *Lactobacillus gasseri* | ATCC 9857 | MRSA |
| *Lactobacillus jensenii* | ATCC 25258 | MRSA |
| *Lactobacillus vaginalis* | ATCC 49540 | MRSA |
| *Lactobacillus rhamnosus* | IGraM 052 | MRSA |
| *Lactobacillus oris* | IGraM 123 | MRSA |
| *Lactobacillus salivarum* | IGraM 097 | MRSA |
